# 

**Published:** 1887-11

**Authors:** 


### BOOK NOTICES.

N. W. Ayer & Son's American Newspaper Annual for 1887.—This is the completest book of its class which has ever been brought to our notice; it is, in fact, a cyclopedia of general information respecting every county in the United States and the Canadian Provinces, compiled with great care from the latest reliable data. As a ready reference, it is invaluable, and shows no spite towards such newspapers and periodicals as decline the use of its columns wherein to advertise, by underating their circulation after the manner of a New York competitor. Published by N. W. Ayer & Son, Philadelphia. Price $3.

Problems of the Day, by Dr. R. C. Flower. This is a pamphlet of fifty-two pages, issued by the Spectator Publishing Company, Boston, Mass. The subjects treated of are "Physical and Mental Degeneracy; The Problem of Life, and Evils of the Bar Room." There is much information in this little volume, and many hints of value to individuals and families, which, if followed, can be productive only of good.

The Invalid's Visitor, edited by Mrs. Kate Sumner Burr, Williamson, N. Y., is the organ of an association whose object is to relieve the weariness of the sick room, by christian and charitable means. May it prosper in good works.

The Fifteenth Annual Announcement of the Physio-Medical College of Indiana, is before us, showing this institution, which aims "to know the truth and adhere to it, to understand the law and be governed by it," is in a healthy and prosperous condition, as it certainly ought to be.

Ovarian Tumors.—We have received a copy of the admirable illustrated lectures of Prof. Edward Borck, A. M. M. D., of St. Louis, upon the above theme, and we fully agree with our professional brethren as to their merit.

The Oread is the official organ of Mount Carroll Seminary, for young ladies, Carroll Co., Ill. This particular Oread, like little Jackey Horner, after eating his christmas pie, is full of nice things, for you can put your thumb in almost anywhere and pull out a plum. Quite a number of Mount Carroll nymphs have been pulled out, too, and it is said they make excellent wives.

Vick's Illustrated Monthly.—We have so often commended this beautifully illustrated horticultural publication, that our readers have grown familiar with our praises of it. All we want to say now is, that we stick to what we said before. The October number is a proof that we are right. Published by James Vick, Seedsman, Rochester. N. Y.

American Otological Society.—The twelfth annual report of this body is one of unusual interest. It is indeed, commendable, that so many eminent specialists as recently assembled at New London, Conn., were drawn together to compare notes and experiences of their several practices in restoring impaired hearing, and healing diseases which threaten the loss of so important a sense. You have our thanks, gentlemen, for the published account of your proceedings replete with valuable information.

Drift, Vol. 1. No., 1., is the pioneer number of an illustrated sheet issued by Drift Publishing Co., Buffalo, N. Y. once a month. Its contents are humorous and quite original.

The Health Record, Corning, N. Y.—First organ of the movement Cure in America we now number among our exchanges. This is an able exponent of the above system of cure, amply illustrated.

